# Trends in Fentanyl Dispensing in Spain: The Case of Galicia (2019–2025)

**DOI:** 10.3390/diseases14070236

**Published:** 2026-06-30

**Authors:** Severo Vázquez-Prieto, Antonio Vaamonde Liste

**Affiliations:** 1 Facultad de Ciencias de la Salud, Universidad Tecnológica Atlántico-Mediterráneo—UTAMED, 29590 Malaga, Spain; 2 Departamento de Estadística e Investigación Operativa, Facultad de Ciencias Económicas y Empresariales, Universidad de Vigo, 36310 Vigo, Spain; vaamonde@uvigo.gal

**Keywords:** drug utilization study, defined daily dose, opioid prescription, fentanyl, administration routes, demographic and socioeconomic factors

## Abstract

In recent years, numerous countries have recorded a steady increase in opioid use. Although prescribing practices vary considerably among them, fentanyl is among the most frequently prescribed strong opioids in several European countries and in Spain. In this study, we analyzed the evolution of outpatient fentanyl dispensing in Galicia, Spain, between January 2019 and December 2025, using the Anatomical Therapeutic Chemical Classification/Defined Daily Dose (ATC/DDD) system. We paid particular attention to differences between provinces and explored temporal trends broken down by route of administration (buccal, nasal, sublingual and transdermal). Dispensing data were obtained from the General Sub-directorate of Pharmacy of the Galician Health Service (SERGAS) from the monthly billing database of official prescriptions dispensed in Galician pharmacies and were expressed as defined daily dose per 1000 inhabitants per day (DID). Dispensing rates between provinces were compared using the non-parametric Kruskal–Wallis test, considering a *p*-value less than 0.05 as statistically significant. We observed an increase in fentanyl use between 2019 and 2021, followed by a systematic decrease during the 2022–2025 period. Significant differences (*p* < 0.001) were found in the defined daily dose per 1000 inhabitants per day (DID) among the four Galician provinces, and demographic and socioeconomic factors partially explain the observed disparities. Regarding pharmaceutical presentations, transdermal patches were the most frequently used form during the study period. While some limitations should be noted, the results suggest that the observed decrease in fentanyl dispensing in Galicia could be associated with the implementation of the Ministry of Health’s 2021 optimization plan, with a sustained reduction in its dispensing from 2022 onwards. However, it is necessary to maintain pharmacovigilance at the provincial level.

## 1. Introduction

Opioids exert their analgesic effects by binding to receptors in the central and peripheral nervous system, modulating pain perception and reducing the transmission of nociceptive signals [1]. Although prolonged use can lead to tolerance, dependence, or opioid abuse disorder, they are commonly used to treat severe acute pain and moderate-to-severe chronic pain that does not respond to other medications [2].

Over the past decade, opioid use has increased in many countries [3,4,5]. In Spain, although historically this use has been lower than in other European countries, a significant increase has also been observed [6]. Thus, the 2017 report by the Spanish Agency for Medicines and Health Products (AEMPS) quantified an 83% increase in national opioid consumption between 2008 and 2015, rising from 7.25 to 13.1 defined daily doses per 1000 inhabitants per day (DID) [7]. This rapid increase highlights the need for greater monitoring of prescribing patterns.

Although prescribing patterns differ widely between countries [2], fentanyl is the most commonly used opioid analgesic in many of them [3,5,8]. In Spain, fentanyl accounted for 17.51% of total opioid consumption in 2015, increasing from 1.43 DID in 2008 to 2.33 DID in 2015. The most commonly used form was transdermal fentanyl, which accounted for 84% of total fentanyl consumption [7]. Fentanyl is available in multiple formulations with distinct pharmacokinetic profiles: while sublingual tablets and oral transmucosal absorption formulations devices reach plasma concentrations in 15–30 min, transdermal patches release the active principle continuously for 48–72 h, which determines both their clinical indication and their potential for abuse. Currently, there are two indications for fentanyl prescription: the treatment of chronic cancer pain with sustained-release transdermal patches in patients on opioid maintenance therapy and the management of breakthrough cancer pain with immediate-release forms (nasal and buccal absorption forms), which carry a higher risk of addiction [6,9,10].

Although opioid use, including fentanyl, has been studied in several countries over the past few decades [2,3,4,5,8,11], few studies have focused on describing how prescription rates vary at the regional or provincial level. For example, Oliva et al. assessed prescription opioid use at the municipal and health area levels on the mostly rural island of La Gomera (Spain) between 2016 and 2019 [12], while Mordecai et al. analyzed patterns of regional variation in opioid prescribing in primary care in England between 2010 and 2014 [13].

Given the clinical relevance of fentanyl for pain management [14], this study focused on examining its outpatient use in Galicia, Spain, assessing differences between provinces and investigating temporal trends from January 2019 to December 2025. Analyzing fentanyl dispensing patterns at the provincial level within the same autonomous community can be potentially useful for identifying geographical heterogeneity in prescribing patterns for regional pharmaceutical policy, pharmacovigilance, and resource allocation

## 2. Materials and Methods

We conducted a study on fentanyl dispensing among outpatients in Galicia, one of the 17 Spanish autonomous communities, which is divided into four provinces: Pontevedra, Ourense, Lugo, and A Coruña. Dispensing was quantified using the Anatomical Therapeutic Chemical Classification/Defined Daily Dose (ATC/DDD) system, which assigns a DDD to each drug as an international technical unit of measurement. This approach allows for comparison of population exposure to a drug regardless of the packaging system or individually prescribed doses, facilitating trend analysis and interregional comparisons [15]. The analysis focused on the ATC subgroup N02AB03 (fentanyl). Dispensing data were obtained from the General Sub-directorate of Pharmacy of the Galician Health Service (SERGAS) from the monthly billing database of official prescriptions dispensed in Galician pharmacies during the period between January 2019 and December 2025. The data were broken down by route of administration (buccal, nasal, sublingual, and transdermal). Dispensing data were expressed in DID, according to the following formula:

$$DID=\frac{nºDDD\times 1000}{Population\times t}$$

where *nº DDD* represents the total number of *DDDs*, *t* corresponds to the number of days in the month, and *population* is the number of inhabitants in each province associated with the year the medication was dispensed. Annual population data were downloaded from a publicly accessible demographic database of the Galician Institute of Statistics [16]. Expressing dispensing in DID helps minimize differences arising from the different commercial presentation formats and allows the construction of comparable time series, as has been applied in previous studies by this group on antiparasitic and antifungal drugs in the same region [17,18,19,20].

For data processing, a dataset was created in Excel. Statistical analysis was performed using the free statistical software R, version 4.5.2. The comparison of dispensing rates between provinces was performed using the non-parametric Kruskal-Wallis test. The units of observation corresponded to the monthly DID values. The number of observations included in each comparison was 336. The non-parametric method was used after verifying that the data did not follow a normal distribution. A *p*-value less than 0.05 was considered statistically significant.

## 3. Results and Discussion

In the present study, we hypothesized that differences in population aging and rurality levels among the Galician provinces could explain some of the observed variability in fentanyl dispensing. We also expected to detect a possible temporal inflection point linked both to the health disruptions of the COVID-19 pandemic and to the regulatory changes of the 2021 optimization measures plan, one of whose objetives was to modify the prescription pattern of potent opioids in primary care [21].

**Figure 1:**
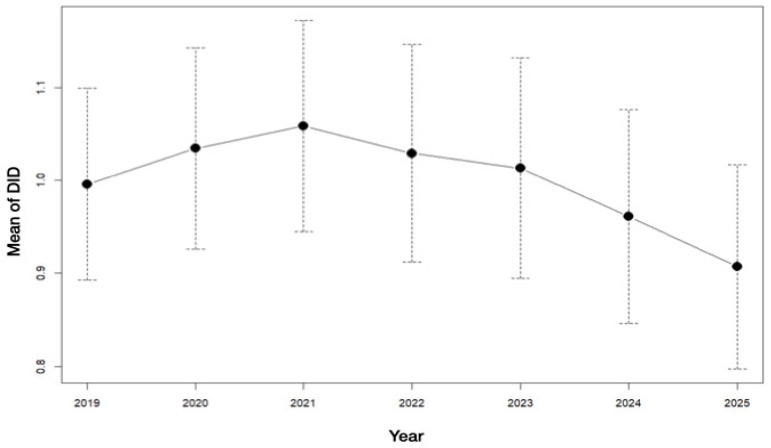
Annual evolution of the defined daily dose per 1000 inhabitants per day (DID) from January 2019 to December 2025 in Galicia (Spain). Error bars around means give plus or minus one standard error of the mean.

Data from Galician community pharmacies between January 2019 and December 2025 reveal a period of sustained increase in total fentanyl dispensing, which peaked in 2021, followed by a phase of gradual decline that extended until the end of the study period, with a particularly pronounced drop in immediate-release formulations (Figure 1). This pattern is consistent with national trends in fentanyl consumption, measured in DIDs (2.70 in 2019, 2.75 in 2020, 2.77 in 2021, 2.68 in 2022, 2.61 in 2023 and 0.51 in 2024) and partially with observations made in other Spanish regions. For example, the prevalence of fentanyl use in primary care in Salamanca doubled between 2011 and 2022, rising from 1.21 DID to 2.56 DID [6]. Similarly, a trend analysis from 2020 to 2023 showed that fentanyl use in Valladolid increased by 56.98%, from 193,674 DDD in 2020 to 304,032 DDD in 2023. However, direct comparisons between regions or time periods are difficult due to methodological variability among studies [22].

Several factors may explain the increase in dispensing between 2019 and 2021. One of these could be the progressive consolidation of fentanyl as the reference opioid for the treatment of chronic pain in Europe [2,5], a development facilitated by its versatility in formulation and the clinical perception of greater gastrointestinal tolerability [23,24,25]. This result should also be interpreted in light of the possible influence of the COVID-19 pandemic, as the healthcare system suffered significant disruptions during that period, which may have affected both access to medical care and prescribing practices. During the pandemic, the suspension or reduction of pain management, physiotherapy, and rehabilitation units may have led to greater reliance on pharmacological analgesia [22,26]. The decrease observed from 2022 onward may be related to the impact of the publication, a year earlier, by the Ministry of Health, of a plan to optimize the use of opioid analgesics for chronic non-cancer pain within the national health system [15], although the observational design of the study does not allow for establishing causal relationships and the observed associations should be interpreted with caution. Furthermore, the gradual reopening of health services beginning in 2022 may have facilitated the reintroduction of multimodal approaches to chronic pain [22,26]. Additionally, the recognition of the potential for dependence and abuse associated with rapid-acting fentanyl formulations may have reinforced more cautious prescribing practices, contributing to the decline. 

**Figure 2:**
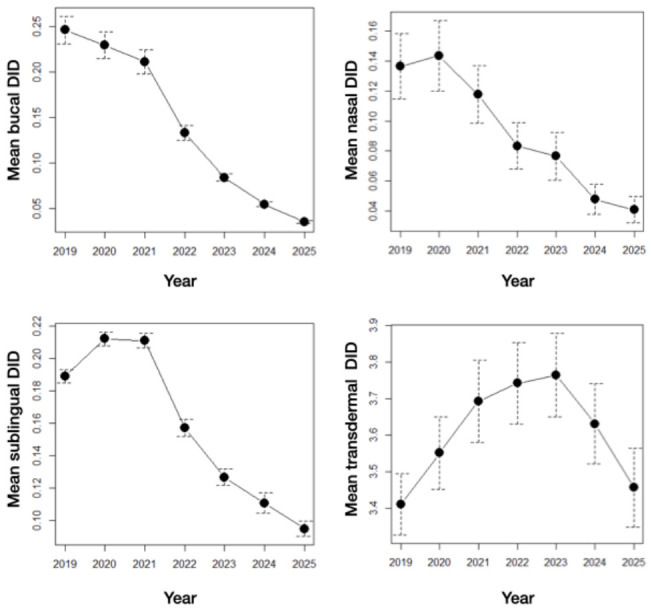
Annual evolution of the defined daily dose per 1000 inhabitants per day (DID) according to the route of administration, from January 2019 to December 2025 in Galicia (Spain). Error bars around means give plus or minus one standard error of the mean.

The trend over time was not the same for each of the four administration methods routes, as can be seen in Figure 2. Analysis of year-over-year variation indicated a slight increase in nasal fentanyl use in 2020, followed by a downward trend. A similar trend was observed for sublingual administration, although in this case it was characterized by a transient increase that lasted a couple of years (2020–2021) before declining. Buccal administration decreased throughout the study period. Regarding transdermal administration, an upward trend in its use was evident between 2019 and 2023, followed by a decrease in 2024 and 2025.

Mean DID differed significantly across the four routes of administration (*p* < 0.001). DID values were clearly higher, both mean and median, for transdermal fentanyl than for immediate-release formulations, which are associated with a higher risk of addiction (Table 1).

The predominance of transdermal fentanyl is consistent with the findings of Torres-Bueno et al., who revealed that this route was also the predominant mode of administration, followed by buccal, sublingual, and nasal forms in Salamanca [6], where transdermal fentanyl patches had already been identified as the most used pharmaceutical form during the period 2000–2006, representing between 80% and 90% of the total [27]. Similarly, on the island of La Gomera, the most frequently prescribed pharmaceutical forms during the study period (2016–2019) were transmucosal lozenges and patches, while the use of fentanyl in nasal spray was low, at 1.31% in 2019 compared to 8.5% in 2017 [12]. In primary care in Catalonia, Perell-Bratescu et al. found a predominance of transdermal fentanyl prescriptions, with a 63.5% increase in DID between 2013 (0.453) and 2017 (0.741) [28]. This preference may reflect a combination of practical and pharmacokinetic factors. Its application and changing schedule every 48 to 72 h facilitate adherence in elderly patients or those with cognitive impairment [29]. Furthermore, it has a lower incidence of gastrointestinal side effects compared to long-acting oral opioids [23,24,25], a feature highly valued by patients.

The results revealed statistically significant differences between the mean DID values in the four Galician provinces (*p* < 0.001), with clearly higher values in the province of Ourense (0.273) compared to Pontevedra (0.135), Lugo (0.126), and A Coruña (0.107). The differences observed between provinces suggest the existence of structural determinants of prescription. Aging is a predisposing factor for the development of chronic musculoskeletal pathologies in which opioid use to treat pain is very common [30]. Galicia is characterized by a high aging index, with more than a quarter of its population over 65 years of age. This population is not evenly distributed. Ourense and Lugo have the highest aging rates, both exceeding 30% of their population over 65 years of age (32.26% and 30.25%, respectively). In contrast, the Atlantic provinces have a slightly less aged demographic structure (26.28% and 24.72% in A Coruña and Pontevedra, respectively), although their aging rate has increased significantly in recent years [31].

Previous studies have suggested that rural areas may have higher rates of opioid prescriptions than urban areas [32,33,34]. The rurality index in Galicia varies significantly, with Lugo and Ourense being the most rural provinces, where access to non-pharmacological alternatives is often limited by geographical dispersion and a scarcity of specialized resources. Conversely, A Coruña and Pontevedra have a higher population density and a more urban/industrial character, especially along the Atlantic coast, although they maintain a network of small rural municipalities, particularly in the inland areas [35]. Furthermore, prescription fentanyl use appears to be more common among people of low socioeconomic status [13]. According to average per capita income indicators, A Coruña is the province with the highest average income in Galicia, followed by Pontevedra, Lugo, and Ourense [36]. Other factors, such as prescribing practices in primary and specialized care and the prevalence of chronic pain, could also be related to the observed geographical variability.

The results are therefore partially consistent with expectations. Ourense’s aging index (32.26% of the population over 65 years of age) and its markedly rural character [31,35] create a demographic profile associated in the literature with a higher prevalence of chronic musculoskeletal pain and, consequently, with greater consumption of potent opioids [30]. However, Pontevedra’s higher DID score compared to Lugo, despite its lower aging index, could be explained by the concentration of specialized hospital resources in the Vigo health area, which attracts patients from neighboring areas. This phenomenon should be explored in future studies using data at the health area level. Furthermore, the accelerated aging of the population in Pontevedra during the period between January 2019 and December 2025 may have increased the demand for opioid analgesia more than anticipated [31]. Finally, it should be noted that the analyses presented here should be considered exploratory and hypothesis-generating. Given that formal statistical evaluation is limited by the small number of provinces (and, therefore, any correlation will have very little statistical power), future work should include a thorough analysis of demographic and socioeconomic factors to provide more conclusive evidence.

This study has other limitations inherent in its design. The DDD is a technical unit that does not always reflect the current prescribed dose or patient adherence, so dispensed medications are not necessary consumed [15]. The lack of data on private prescriptions introduces a systematic underestimation that cannot be accurately determined without access to such records; however, it is presumed to be small given the broad coverage of the national health system. It is also important to note that our dataset was limited to fentanyl, so we cannot directly determine whether the observed pattern was specific to this drug or whether it may reflect a broader trend in the prescribing of other opioids. Consequently, future studies should also evaluate other major opioid analgesics, such as tramadol and tapentadol. Furthermore, illicitly acquired fentanyl is not recorded in pharmacy dispensing databases. Therefore, our findings describe patterns of legal outpatient dispensing and should not be interpreted as representative of total fentanyl exposure in the population. Additional contextual information on the burden of chronic pain, cancer pain, palliative care needs, or other epidemiological indicators would have improved the interpretation of the results; however, while it was also not possible to assess therapeutic appropriateness due to a lack of individual clinical data (diagnosis, current prescribed dose, and duration of treatment), the analysis remains useful for characterizing prescription practices in the studied context. Therefore, linking dispensing records with the SERGAS electronic health record would be a valuable aspect to consider in the next study.

## 4. Conclusions

An increase in fentanyl dispensing was observed between 2019 and 2021, followed by a systematic decrease during the 2022–2025 period. Differences were also observed among the four Galician provinces. Regarding pharmaceutical presentations, fentanyl transdermal patches were the most frequently used form during the study period. While the study has several limitations, these findings help characterize the provincial dynamics of fentanyl use in Galicia and support hypotheses about the factors that modulate it. Linking dispensing data with individualized clinical records from SERGAS would allow for the establishment of causal relationships between prescribing patterns, health outcomes, and patients’ socioeconomic conditions. This could contribute to the development of more specific strategies to optimize pain management, reduce the risk of abuse and dependence, and have direct implications for regional pharmaceutical policy.

## Figures and Tables

**Table 1:** Mean and median values of the defined daily dose per 1000 inhabitants per day (DID) according to the route of administration (n = 336).

Administration Route	Buccal Fentanyl	Nasal Fentanyl	Sublingual Fentanyl	Transdermal Fentanyl
Mean	0.142	0.092	0.157	3.608
Standar deviation	0.106	0.125	0.056	0.741

## Data Availability

The raw data supporting the conclusions of this article will be made available by the authors on request.

## References

[1] Dowell D., Ragan K.R., Jones C.M., Baldwin G.T., Chou R. (2022). CDC Clinical Practice Guideline for Prescribing Opioids for Pain—United States, 2022. MMWR Recomm. Rep..

[2] Bosetti C., Santucci C., Radrezza S., Erthal J., Berterame S., Corli O. (2018). Trends in the consumption of opioids for the treatment of severe pain in Europe, 1990–2016. Eur. J. Pain.

[3] Hamunen K., Paakkari P., Kalso E. (2009). Trends in opioid consumption in the Nordic countries 2002–2006. Eur. J. Pain.

[4] Hastie B.A., Gilson A.M., Maurer M.A., Cleary J.F. (2014). An Examination of Global and Regional Opioid Consumption Trends 1980–2011. J. Pain Palliat. Care Pharmacother..

[5] Hider-Mlynarz K., Cavalié P., Maison P. (2018). Trends in analgesic consumption in France over the last 10 years and comparison of patterns across Europe. Br. J. Clin. Pharmacol..

[6] Torres-Bueno C., Sanchez-Barba M., Miron-Canelo J.-A., Gonzalez-Nunez V. (2024). Evolution of Fentanyl Prescription Patterns and Administration Routes in Primary Care in Salamanca, Spain: A Comprehensive Analysis from 2011 to 2022. Healthcare.

[7] Agencia Española de Medicamentos y Productos Sanitarios Utilización de Medicamentos Opioides en España Durante el Pe-riodo 2008–2015. Madrid: AEMPS; 2017. Informe de Utilización de Medicamentos U/OPI/V1/13022017. https://www.aemps.gob.es/medicamentosUsoHumano/observatorio/docs/opioides-2008-2015.pdf.

[8] del Pozo J.G., Carvajal A., Viloria J.M., Velasco A., del Pozo V.G. (2007). Trends in the consumption of opioid analgesics in Spain. Higher increases as fentanyl replaces morphine. Eur. J. Clin. Pharmacol..

[9] McWilliams K., Fallon M. (2013). Fast-acting fentanyl preparations and pain management. Qjm Int. J. Med..

[10] Brząkała J., Leppert W. (2019). The role of rapid onset fentanyl products in the management of breakthrough pain in cancer patients. Pharmacol. Rep..

[11] Zhu W., Chernew M.E., Sherry T.B., Maestas N. (2019). Initial Opioid Prescriptions among U.S. Commercially Insured Patients, 2012–2017. N. Engl. J. Med..

[12] Mordecai L., Reynolds C., Donaldson L.J., de C Williams A.C. (2018). Patterns of regional variation of opioid prescribing in primary care in England: A retrospective observational study. Br. J. Gen. Pr..

[13] Oliva A., Armas N., Dévora S., Abdala S. (2021). Opioid use trends in Spain: The case of the island of La Gomera (2016–2019). Naunyn-Schmiedebergs Arch. Pharmacol..

[14] Kuhn G.P., Bigal A.L., Nappo S.A. (2025). Fentanyl: A threat to Brazilian society or an opioid drug of great importance in pain management?. Saude E Soc..

[15] Laporte J.R., Tognoni G. (1993). Estudios de utilización de medicamentos y farmacovigilancia. Principios de Epidemiología del Medicamento.

[16] Instituto Gallego de Estadística. https://www.ige.eu/igebdt/selector.jsp?COD=590&paxina=001&c=0201001002.

[17] Prieto S.V., Vaamonde A., Paniagua E. (2022). Study of the Use of Antinematode Drugs, Mebendazole and Pyrantel, in Galicia (Spain) from 2016 to 2020. J. Parasitol. Res..

[18] Vázquez-Prieto S., Vaamonde A., Paniagua E. (2023). Study of the Use of Permethrin 5% Cream in Galicia (Spain) between 2018 and 2021. Infect. Dis. Rep..

[19] Vázquez-Prieto S., Vaamonde A., Paniagua E. (2024). An Analysis of the Use of Systemic Antifungals (Fluconazole, Itraconazole, and Terbinafine) in Galicia, Spain, between 2019 and 2022. Diseases.

[20] Vazquez-Prieto S., Vaamonde A., Paniagua E. (2025). Retrospective Analysis of the Use of Oral Ivermectin and 5% Permethrin Cream in Galicia (Spain). Basic Clin. Pharmacol. Toxicol..

[21] (2021). Plan de Optimización de la Utilización de Analgésicos Opioides en dolor Crónico no Oncológico en el Sistema Nacional de Salud. https://www.sanidad.gob.es/areas/farmacia/publicaciones/planOptimizacion/docs/opioides/Plan_Optimizacion_Opioides_en_DCNO.pdf.

[22] Enríquez de Salamanca Gambara R., Sierra Santos A.M., Ruiz San Pedro A.M., Montero Cuadrado F., Muñoz León I., Castro Villamor M.Á., Córdoba Romero A., Del Olmo Tornero A.M., Pérez Pérez L., Morales-Quezada L. (2025). Prescription of Strong Opioids in Chronic Non-Cancer Pain in the Province of Valladolid (Spain). Life.

[23] Radbruch L., Sabatowski R., Loick G., Kulbe C., Kasper M., Grond S., A Lehmann K. (2000). Constipation and the use of laxatives: A comparison between transdermal fentanyl and oral morphine. Palliat. Med..

[24] Tassinari D., Sartori S., Tamburini E., Scarpi E., Tombesi P., Santelmo C., Maltoni M. (2009). Transdermal Fentanyl as A Front-Line Approach to Moderate-Severe Pain: A meta-analysis of Randomized Clinical Trials. J. Palliat. Care.

[25] Tassinari D., Sartori S., Tamburini E., Scarpi E., Raffaeli W., Tombesi P., Maltoni M. (2008). Adverse Effects of Transdermal Opiates Treating Moderate-Severe Cancer Pain in Comparison to Long-Acting Morphine: A Meta-Analysis and Systematic Review of the Literature. J. Palliat. Med..

[26] Marinangeli F., Giarratano A., Petrini F. (2020). Chronic Pain and COVID-19: Pathophysiological, clinical and organizational issues. Minerva Anestesiol..

[27] Díaz Madero A., Ramos Pollo D., Martín González M. (2009). Evolución del consumo de opioides en Castilla y León desde el año 2000 al 2006. Med. Paliativa.

[28] Perelló-Bratescu A., Dürsteler C., Álvarez-Carrera M.A., Granés L., Kostov B., Sisó-Almirall A. (2022). Trends in the Prescription of Strong Opioids for Chronic Non-Cancer Pain in Primary Care in Catalonia: Opicat-Padris-Project. Pharmaceutics.

[29] Reddy A., Tayjasanant S., Haider A., Heung Y., Wu J., Liu D., Yennurajalingam S., Reddy S., de la Cruz M., Rodriguez E.M. (2015). The opioid rotation ratio of strong opioids to transdermal fentanyl in cancer patients. Cancer.

[30] Huang Y.-L., Tsay W.-I., Her S.-H., Ho C.-H., Tsai K.-T., Hsu C.-C., Wang J.-J., Huang C.-C. (2020). Chronic pain and use of analgesics in the elderly: A nationwide population-based study. Arch. Med. Sci..

[31] Principales Resultados: Indicadores de Población. Galicia y Provincias. Año 2025. Instituto Gallego de Estadística. https://www.ige.gal/igebdt/principais.jsp?COD=722&R=1[14:15:0:10:11:12:4:5:6:7:8:16:9:17]&C=9912[all]&F=T[1:0];2:0.

[32] Keyes K.M., Cerd M., Brady J.E., Havens J.R., Galea S. (2014). Understanding the rural-urban differences in nonmedical prescription opioid use and abuse in the United States. Am. J. Public Health.

[33] Sears J.M., Edmonds A.T., Fulton-Kehoe D. (2019). Tracking Opioid Prescribing Metrics in Washington State (2012-2017): Differences by County-Level Urban-Rural and Economic Distress Classifications. J. Rural Health.

[34] Shoff C., Yang T., Kim S. (2020). Rural/Urban Differences in the Predictors of Opioid Prescribing Rates Among Medicare Part D Beneficiaries 65 Years of Age and Older. J. Rural Health.

[35] Panorama Rural-Urbano. Instituto Gallego de Estadística. https://www.ige.gal/web/mostrar_actividade_estatistica.jsp?idioma=es&codigo=0701.

[36] Atlas de Distribución de Renta de los Hogares. Instituto Nacional de Estadística. https://www.ine.es/dyngs/INEbase/es/operacion.htm?c=Estadistica_C&cid=1254736177088&menu=ultiDatos&idp=1254735976608.

